# Unraveling the Role of Reactive Oxygen Species in T Lymphocyte Signaling

**DOI:** 10.3390/ijms25116114

**Published:** 2024-06-01

**Authors:** Karsten Gülow, Deniz Tümen, Philipp Heumann, Stephan Schmid, Arne Kandulski, Martina Müller, Claudia Kunst

**Affiliations:** Department of Internal Medicine I, Gastroenterology, Hepatology, Endocrinology, Rheumatology, Immunology, and Infectious Diseases, University Hospital Regensburg, 93053 Regensburg, Germany; deniz.tuemen@ukr.de (D.T.); philipp.heumann@klinik.uni-regensburg.de (P.H.); stephan.schmid@ukr.de (S.S.); arne.kandulski@ukr.de (A.K.); martina.mueller-schilling@ukr.de (M.M.); claudia.kunst@ukr.de (C.K.)

**Keywords:** T lymphocytes, reactive oxygen species (ROS), oxidative signal, T cell activation, T cell receptor (TCR), electron transport chain (ETC), glycolysis, metabolic shift

## Abstract

Reactive oxygen species (ROS) are central to inter- and intracellular signaling. Their localized and transient effects are due to their short half-life, especially when generated in controlled amounts. Upon T cell receptor (TCR) activation, regulated ROS signaling is primarily initiated by complexes I and III of the electron transport chain (ETC). Subsequent ROS production triggers the activation of nicotinamide adenine dinucleotide phosphate oxidase 2 (NADPH oxidase 2), prolonging the oxidative signal. This signal then engages kinase signaling cascades such as the mitogen-activated protein kinase (MAPK) pathway and increases the activity of REDOX-sensitive transcription factors such as nuclear factor-kappa B (NF-κB) and activator protein-1 (AP-1). To limit ROS overproduction and prevent oxidative stress, nuclear factor erythroid 2-related factor 2 (Nrf2) and antioxidant proteins such as superoxide dismutases (SODs) finely regulate signal intensity and are capable of terminating the oxidative signal when needed. Thus, oxidative signals, such as T cell activation, are well-controlled and critical for cellular communication.

## 1. Introduction

Prior to the emergence of photosynthetic archaebacteria approximately 2.7 billion years ago, the Earth’s atmosphere was predominantly anoxic, lacking significant levels of oxygen. At that time, life on Earth was completely anaerobic and, thus, the advent of oxygen (O_2_) generated by oxygenic photosynthesis posed a significant evolutionary challenge to terrestrial life forms on Earth. The absence of defense mechanisms against oxidative damage rendered O_2_ toxic to many organisms, precipitating widespread extinctions across numerous species. Nevertheless, life found adaptive pathways for survival and evolved protective mechanisms, eventually harnessing oxygen for energy generation via oxidative phosphorylation [1,2,3,4]. However, managing the utilization of oxygen for energy production posed additional hazards, notably the generation of reactive oxygen species (ROS). Once again, life adapted and evolved complex systems to build a defense against these highly reactive toxic agents [5,6,7].

Over time, reactive oxygen species (ROS) have transitioned from being undesired byproducts of enzymatic reactions to molecules intentionally produced and by regulation, serving as signaling molecules or weapons to eliminate pathogens [8,9,10]. To comprehend the utilization of ROS as signaling molecules, one should (i) be familiar with the chemical properties of the major ROS, (ii) understand the key mechanisms governing their control and removal, and (iii) be aware of the primary sources of ROS production in eukaryotic cells.

### 1.1. Key ROS Vital for T Cell Signaling

The primary ROS crucial for cellular signaling include superoxide anions (O_2_^•−^), hydroxyl radicals (^•^OH), and hydrogen peroxide (H_2_O_2_) [8,9,10,11,12]. Cellular ROS production typically begins with electron transfer to O_2_, forming O_2_^•−^. Due to their energetically unstable state, these molecules exhibit high reactivity and possess a short half-life of approximately 1 µs. Furthermore, their charged nature impedes free diffusion through cellular membranes. Consequently, O_2_^•−^ exerts a locally constrained impact, primarily contributing to oxidative damage rather than serving as a signaling molecule [9,10].

In an aqueous environment, O_2_^•−^ rapidly transforms into H_2_O_2_. Intracellularly, this conversion is expedited by superoxide dismutases (SODs) [10,13,14,15]. Despite H_2_O_2_ not being a radical, it is classified as an ROS. H_2_O_2_ exhibits a longer half-life of approximately 1 ms compared to O_2_^•−^. Moreover, H_2_O_2_ can freely diffuse through membranes, and it primarily targets free thiols. This oxidation is generally reversible. Thus, H_2_O_2_ meets the criteria to serve as a second messenger [9,10,16,17].

However, an accumulation of H_2_O_2_ also poses risks of cellular damage. To ensure proper signaling, concentrations of H_2_O_2_ must be maintained within a specific range. Additionally, an accumulation of H_2_O_2_ increases the likelihood of its interaction with free iron^2+^ (Fe^2+^). While Fe^2+^ generally exists in a bound form in cells, a small free fraction exists called the labile iron pool. When H_2_O_2_ encounters free Fe^2+^, highly reactive hydroxyl radicals (^•^OH) are generated. These radicals have a half-life of less than 1 µs and can lead to massive cell damage, including lipid peroxidation [9,10,18,19].

### 1.2. The Essential Components of the Oxidative Defense

To prevent the accumulation of ROS, a complex network of antioxidative enzymes and ROS scavengers exists [8,10]. Superoxide dismutases (SODs) are essential enzymes that catalyze the conversion of O_2_^•−^ into H_2_O_2_. SODs play a vital role in the disproportionation of O_2_^•−^ both intracellularly and extracellularly, thus safeguarding cell membranes and DNA from ROS-mediated damage. In the cytosol and mitochondrial intermembrane space, SOD1 catalyzes the conversion of O_2_^•−^ into H_2_O_2_; in the mitochondrial matrix, SOD2 performs this function; and extracellularly, SOD3 fulfills this task [13,20,21].

Catalase eliminates H_2_O_2_, transforming it into O_2_ and water (H_2_O). The chemical equation is 2H_2_O_2_ → O_2_ + 2H_2_O. Catalase is primarily active in peroxisomes, where it neutralizes excessive H_2_O_2_. Catalase can also be extracellularly secreted, where it may associate with the plasma membrane or disperse in the extracellular milieu [22,23,24].

Another important factor in H_2_O_2_ removal is the tripeptide glutathione, which acts as an effective oxidative scavenger in its reduced form (GSH). With concentrations typically ranging from 1 to 10 mM, GSH emerges as the primary antioxidant within cells, playing a pivotal role in the efficient removal of H_2_O_2_ [25]. Abundantly distributed across various cellular compartments, glutathione is present in the cytosol, endoplasmic reticulum (ER), mitochondria, vacuoles, and peroxisomes. The scavenging ability of GSH is attributed to its high reductive potential. A central cysteine residue with nucleophilic characteristics is the source of its reducing power. GSH can directly reduce H_2_O_2_ or reverse thiol oxidations on proteins [8]. In addition to GSH, other thiol scavengers are thioredoxin 1 and 2. Thioredoxins serve as key disulfide (-S-S-) reductases within the cell, primarily reversing thiol oxidations [9,26].

### 1.3. The Sources of ROS Production in T cells

ROS are generated at multiple cellular sites, including the mitochondria, peroxisomes, endoplasmic reticulum (ER), peroxisomes, and plasma membrane. Among these, nicotinamide adenine dinucleotide phosphate oxidases (NADPH oxidases) at the plasma membrane and mitochondria stand out as prominent sites for ROS generation in immune cells [27].

The NOX family comprises inducible NOX complexes 1–5 associated with the plasma membrane and two dual oxidases (DUOX)1/2. NOX2 (respiratory burst NOX), the most extensively studied isoform, is primarily expressed in phagocytes (e.g., at high levels—neutrophils, macrophages, dendritic cells; at low levels—B cells, mast cells, eosinophils, natural killer cells), where it facilitates ROS-dependent pathogen killing [28,29]. It is also found in certain non-phagocytic cell types, including T cells [30,31,32]. NOX2 consists of various components: the transmembrane flavocytochrome b558 (a heterodimer containing gp91phox and p22phox) as well as the four cytoplasmic proteins p47phox, p67phox, p40phox, and small G protein Rac1/2. Upon activation, the NOX2 complex generates membrane-impermeant O_2_^•−^ towards the inside of the phagosomes or the extracellular side of the plasma membrane. There, O_2_^•−^ can then be converted into H_2_O_2_ and re-enter the cell to work as a second messenger [9,29].

Aerobic respiration leads to substantial ROS production by mitochondria. Mitochondrial ROS are primarily released at two sites within the electron transport chain (ETC): Complex I (NADH dehydrogenase) and Complex III (ubiquinone-cytochrome c reductase) [10,29,33,34]. Mitochondrial ROS serve as crucial signals in various cellular processes such as adaptation to hypoxia, regulation of innate or adaptive immunity, differentiation, or autophagy [10,27,29,35,36]. Mitochondrial ROS act as important signaling molecules, particularly in T cells, determining the activation and deactivation of T cell-mediated immune responses [29].

In summary, the nature of produced ROS, the cellular oxidative defense, and ROS localization are critical factors determining the induction of the generation of an oxidative signal [10,11]. To enable oxidative signaling, a constant oxidative environment must be established [8].

Oxidative processes, once hazardous in early life’s development, are utilized not only by the majority of organisms for energy production but also for signal transduction. This review focuses on oxidative signaling cascades in T lymphocytes and explores the mechanisms driving their formation.

## 2. T Cell Receptor (TCR) Stimulation-Induced Oxidative Signaling

T cells are an integral part of the adaptive immune response. Like all blood cells, T cells originate in the bone marrow. From there, they migrate to the thymus. In the thymus, T cells undergo differentiation and selection to specifically recognize a particular antigen. Upon exiting the thymus, these cells circulate throughout the body and scan major histocompatibility (MHC) complexes displayed on the surface of antigen-presenting cells (APCs) to locate their target antigen. A target antigen binding to the T cell receptor (TCR) subsequently triggers a complex signaling cascade that initiates T cell activation.

Antioxidants were observed to inhibit T cell activation, suggesting a regulatory role of ROS [37,38,39]. Subsequently, direct evidence of TCR-triggered ROS production is demonstrated in various studies [40,41,42,43,44]. This supports the specific involvement of ROS in T cell activation signaling pathways, emphasizing the intricate role of oxidative processes in cellular responses [29,45,46].

### 2.1. The Proximal TCR Machinery

**Figure 1:**
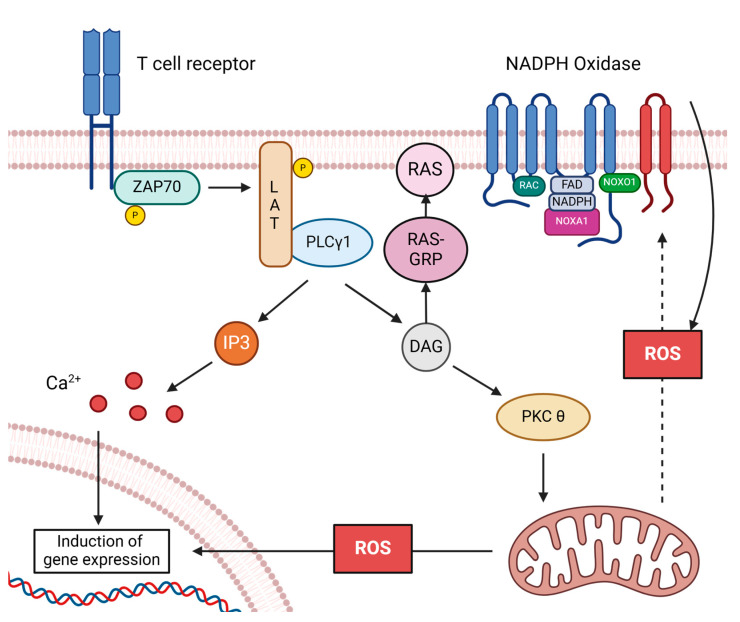
Schematic representation of oxidative signaling in T cells. Stimulation of the T cell receptor (TCR) leads to phosphorylation and activation of tyrosine kinase ZAP70, which phosphorylates LAT. Consequently, LAT recruits PLCγ1, which generates inositol 3,4,5-triphosphate (IP_3_) and diacylglycerol (DAG). At this point, the activation-induced signal diverges. IP_3_ is responsible for releasing Ca^2+^ into the cytosol. DAG activates the RAS guanyl-releasing protein 1 (RAS-GRP) and PKCθ. RAS-GRP activates Rat sarcoma (RAS) and subsequent kinase signaling. PKCθ induces ROS release via the mitochondria. Both signals are essential for the induction of activation-induced gene expression in T cells. Mitochondrial ROS release is a prerequisite for the induction of NADPH oxidase 2. ROS generation via NADPH oxidase 2 leads to a sustained oxidative signal. The figure was created with BioRender.com (accessed on 31 May 2024).

Upon TCR stimulation, receptor-bound tyrosine kinases are activated, including the Zeta-chain-associated protein kinase 70 (ZAP70). ZAP70 subsequently phosphorylates the adapter protein Linker for activation of T cells (LAT), to which Phosphoinositid-phospholipase (PLC)γ1 is recruited and activated [47,48,49]. Activation of PLCγ1 results in the generation of inositol 3,4,5-triphosphate (IP_3_) and diacylglycerol (DAG). IP_3_ increases cytosolic calcium (Ca^2+^) levels, while DAG activates protein kinase C θ (PKCθ). A subpopulation of activated PKCθ is translocated to the mitochondria and induces controlled ROS generation by the ETC [29,32,46,50,51]. Concurrently, activation of NADPH oxidase 2 is induced, ensuring a sustained ROS signal [30]. Thus, mitochondria are crucial for initiating the oxidative signal, while NADPH oxidase 2 is responsible for its maintenance [32] (Figure 1 and Table 1).

Importantly, both signals, Ca^2+^ release and ROS production, constitute the minimal requirement for inducing the expression of essential interleukins crucial for initiating an immune response [29,41,50].

### 2.2. Mitochondria: Oxidative Signaling Hub in TCR Activation

Following TCR stimulation, mitochondria serve as the central oxidative signaling platform. Upon translocation of a subpopulation of PKCθ to the mitochondria, there is a controlled release of O_2_^•−^ into the mitochondrial matrix, mediated by Complex I [29,32,36], and of Complex III of the ETC to the mitochondrial intermembrane space [33,35,51,52,53] (Table 1).

**Figure 2:**
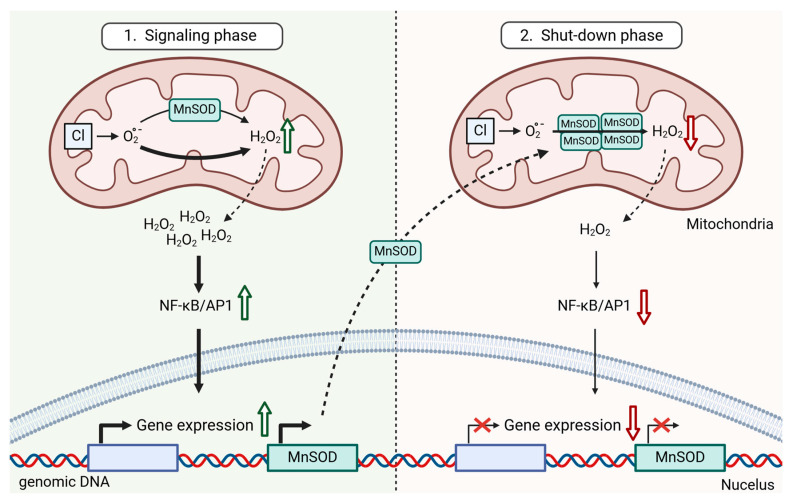
Complex I (CI) releases O_2_^•−^ into the mitochondrial matrix, where most of it is converted to H_2_O_2_. However, certain conditions can lead to increased H_2_O_2_ production through other reactions. This high H_2_O_2_ production induces MnSOD, which further accelerates the conversion of O_2_^•−^ to H_2_O_2_. As a result, fewer alternative reactions leading to H_2_O_2_ formation occur. Overall, this leads to reduced H_2_O_2_ production and a downregulation of the oxidative signal. The figure was created with BioRender.com.

During later phases of TCR signaling, upregulation of MnSOD (SOD2) expression occurs to mitigate potential oxidative damage to the mitochondria. Elevated MnSOD levels lead to reduced oxidative signaling, contributing to the cessation of TCR-triggered T cell activation. The precise mechanism by which MnSOD blocks the oxidative signal remains incompletely understood. It is hypothesized that the high efficiency of MnSOD in scavenging oxygen reduces the probability of oxygen participating in other reactions that could generate H_2_O_2_ (e.g., reduction in the Aconitase [4Fe–4S] cluster) and therefore produces less H_2_O_2_ per oxygen molecule than the non-catalyzed conversion of O_2_^•−^ to H_2_O_2_ [20] (Figure 2). This is in line with two studies emphasizing the critical role of MnSOD in T cell homeostasis. The first study demonstrated that T cell-specific MnSOD knockout increases mitochondrial superoxide anion levels, leading to the hyperactivation of thymocytes and peripheral T cells [54]. The second study shows that reduced MnSOD activity and content in mouse T cells lacking the interferon (IFN)-γ-inducible lysosomal thiol reductase (Gilt^−/−^) result in increased TCR-triggered ERK activation and proliferation by elevated intracellular ROS levels [55].

A study by Sena et al. highlights the role of Complex III as an additional ROS source following TCR stimulation. The authors report the necessity of intact Complex III for T cell activation and mitochondrial ROS production through this complex. Using a T cell-specific knockout mouse with a deletion of the Rieske iron-sulfur protein (RISP) subunit of Complex III, they demonstrated impaired CD4^+^ and CD8^+^ T cell responses in murine models of asthma and listeriosis, respectively [51].

Complex III releases ROS into both the mitochondrial matrix and the mitochondrial intermembrane space. Here, O_2_^•−^ converts into H_2_O_2_ and can act as second messenger in the cytosol. Cu/ZnSOD (SOD1) could play a role similar to MnSOD in the matrix by protecting the cell against oxidative damage and terminating the oxidative signal.

**Table 1:** Overview of key signaling molecules responsible for ROS induction and complexes generating the oxidative signal.

TCR Activation-Induced Oxidative Signaling			
Proximal Proteins of the TCR Machinery Essential for Oxidative Signal Induction			
Protein Name	Function		References Demonstrating the Essential Role of These Proteins in Oxidative Signaling.
Zeta-chain-associated protein kinase 70 (ZAP70)	phosphorylation of LAT	signal cascade from the T cell receptor (TCR) to the mitochondria (essential proteins for the induction of activation-induced oxidative signals)	Kaminski et al., 2007 [32], Kaminski et al., 2010 [50], Kaminski et al., 2012 [36]
Linker for activation of T cells (LAT)	scaffold protein recruiting PLCγ1 upon phosphorylation		
Phosphoinositide-phospholipase 1 (PLCγ1)	PLCγ1 generates inositol 3,4,5-triphosphate (IP_3_) and diacylglycerol (DAG)		
Protein kinase C θ (PKCθ)	a subpopulation DAG-activated PKCθ translocates to mitochondria and induces ROS release		
**Initial Oxidative Signaling: ETC Complexes Releasing ROS upon TCR Triggering**			
**Name of the ETC Complex**	**ROS Is Released Into:**		**References Demonstrating ROS Production via the ETC**
Complex I	the mitochondrial matrix		Kaminski et al., 2007 [32], Kaminski et al., 2010 [50], Kaminski et al., 2012 [36]
Complex II	the mitochondrial intermembrane space		Sena et al., 2013 [51]
**Protein Complexes Involved in Sustained Oxidative Signaling**			
**Name of the Complex**	**ROS Is Released Into:**		**References Demonstrating ROS Production via NADPH Oxidases**
NADPH oxidase 2	the extracellular space		Jackson et al., 2004 [30], Krammer et al., 2007 [56] Kaminski et al. 2007 [32]

## 3. Interplay between Glucose Metabolism and Oxidative Signaling

**Figure 3:**
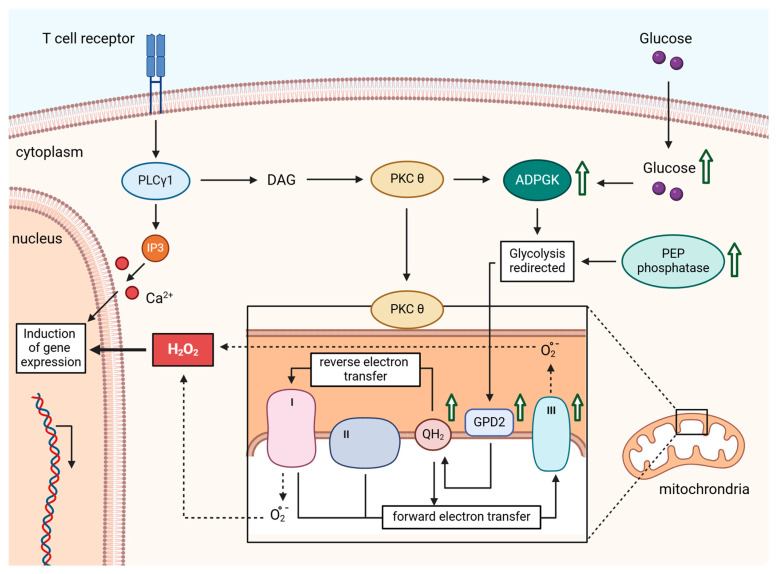
After TCR stimulation, PLCγ1 generates 3,4,5-triphosphate (IP_3_) and diacylglycerol (DAG). IP_3_ induces Ca^2+^ influx into the cytosol and activates NF-AT via calcineurin. Simultaneously, DAG activates PKCθ. PKCθ most likely phosphorylates ADPGK. ADPGK induces a redirection of glycolysis, allowing electrons to be directly transferred to ubiquinone via GPD2. From there, electrons can be retrogradely directed towards Complex I of the ETC and forward directed to Complex III. At these complexes, ROS is released, activating REDOX-sensitive transcription factors and inducing the expression of specific genes essential for T cell activation. The figure was created with BioRender.com.

Mitochondria serve as aerobic energy producers, with the respiratory chain requiring a precise balance between pro-oxidative and anti-oxidative systems for optimal operation. ROS are natural byproducts of this process. However, during the generation of oxidative signals, specific and finely regulated quantities of ROS are deliberately released [29,57]. As described in Section 2, mitochondrial ROS are generated in a regulated manner by Complexes I and III following TCR stimulation. Complex I is the site where electrons from NADH are fed into the respiratory chain. Following TCR stimulation, mitochondrial ROS are primarily generated by Complex I most likely through reverse electron transport (RET). RET occurs in a two-step process involving reduced coenzyme Q (CoQ) and a change in proton motive force that drives electrons back into Complex I, where ROS are released into the mitochondrial matrix [29,32,57,58,59]. Complex III generates ROS as well. Under normal conditions, electrons flow from the CoQ pool to cytochrome C. However, alterations within the ubiquinone binding site of Complex III can prompt O_2_ to react with ubisemiquinone, resulting in O_2_^•−^ formation. O_2_^•−^ generated by Complex III is primarily released into the intermembrane space. After disproportionation, H_2_O_2_ can diffuse freely into the cytosol either from the matrix or from the intermembrane space [60,61] (Figure 3).

A shift in cellular metabolism is essential to modify electron flow, which is accompanied by increased glucose uptake. Only this metabolic adjustment ensures the targeted release of oxidative signals through mitochondria.

### 3.1. Cellular Metabolic Alterations Are Essential for Oxidative Signal Generation

The metabolic program of T cell activation involves a transition from quiescence to increased energy demand upon TCR engagement. Naïve T cells primarily rely on oxidative phosphorylation for ATP production, fueled by pyruvate oxidation and fatty acid oxidation, with low glycolytic activity. TCR engagement leads to activation, proliferation, and the differentiation of naïve T cells into effector, memory, and central memory T cells, accompanied by the upregulation of key metabolic enzymes such as glucose transporter GLUT1 and acetyl-CoA carboxylase 1 (ACC1) [62].

TCR signaling relies on glucose uptake [63,64] and is accompanied by a metabolic shift from mitochondrial ATP production to aerobic glycolysis, comparable to the Warburg effect [65,66,67], a phenomenon characteristic of rapidly proliferating cells [66,68]. Therefore, upon T cell activation, the mitochondrial respiratory chain switches from an ATP-producing to an oxidative signaling function, while glycolysis fulfills cellular energy demands.

In this context, TCR triggering has been shown to activate ADP-dependent glucokinase (ADPGK), an ER-localized glycolytic enzyme [36,69]. ADPGK activation is associated with a rapid glucose uptake, downregulation of mitochondrial oxygen consumption, and redirection of glycolysis via the Glycerin-3-phosphat-Dehydrogenase (GPD) shuttle. Glycerol-3-phosphate dehydrogenase 2 (GPD2), located proximal to the ETC at the inner mitochondrial membrane, oxidizes glycerol-3-phosphate to dihydroxyacetone phosphate. This process directs electrons into the CoQ pool, leading to the generation of ubiquinol and the onset of an electron backlog. This backlog results in RET, with the subsequent release of O_2_^•−^ from Complex I [36,70]. Thus, this process represents the initial step in the generation of the activation-induced oxidative signal (Figure 3).

In addition to Complex I, Complex III also contributes to activation-induced oxidative signaling in T cells. The release of ROS via Complex III has been demonstrated through knockout of the Rieske iron-sulfur protein (RISP), a component of Complex III. RISP knockout can impair both the oxidative signal and the activation of T cells [51,70]. The release of ROS via Complex III may be induced by enhanced electron transport from ubiquinol toward Complex III, leading to electron flow from ubiquinol both backward to Complex I [36] and forward to Complex III (Figure 3). An alternative explanation could involve an increased electron flow through the ETC due to an enhanced production of reducing equivalents NADH and FADH_2_. These could be generated by heightened activity of the tricarboxylic acid cycle (TCA cycle) induced by the activation-induced Ca^2+^ signal [35]. However, the exact mechanism requires further detailed investigation.

The individual complexes of the mitochondrial ETC play distinct roles in generating ROS and regulating cellular functions, such as inflammation, epigenetic changes, and T cell differentiation [71]. A fundamental prerequisite for mitochondria to become oxidative signaling platforms is a metabolic alteration associated with increased glucose uptake, irrespective of whether Complex I, Complex III, or both complexes generate the oxidative signal.

### 3.2. Elevated Glucose Uptake in Different T Cell Subsets

An increase in glucose uptake and a shift from respiration and ATP production via oxidative phosphorylation to aerobic glycolysis is observed in many T cell subsets following TCR stimulation [72]. Elevated glucose uptake induces heightened glycolytic flux, leading to elevated ROS levels. This heightened glycolytic flux typically correlates with increased activity in the TCA cycle, resulting in the accumulation of ETC substrates and elevated NADH/NAD^+^ and FADH2/FAD ratios [73]. Excessive mitochondrial NADH levels promote an elevated electron flow through the ETC, triggering the formation of O_2_^•−^ by Complex III [51]. Additionally, the combination of an increased glycolytic flux, GPD2-dependent hyper-reduction in ubiquinone, and a highly negative membrane potential (Δψ) lead to O_2_^•−^ production by Complex I via RET [29]. The metabolic shift is thus a prerequisite for the generation of the oxidative signal and for T cell activation. To enable the shift toward glycolysis, increased glucose import is required. Only through this metabolic shift can controlled ROS release from Complex I and Complex III occur. This oxidative signal then regulates the activation and differentiation of T cells.

#### 3.2.1. Increased Glucose Uptake in CD4^+^ T Cells

**Figure 4:**
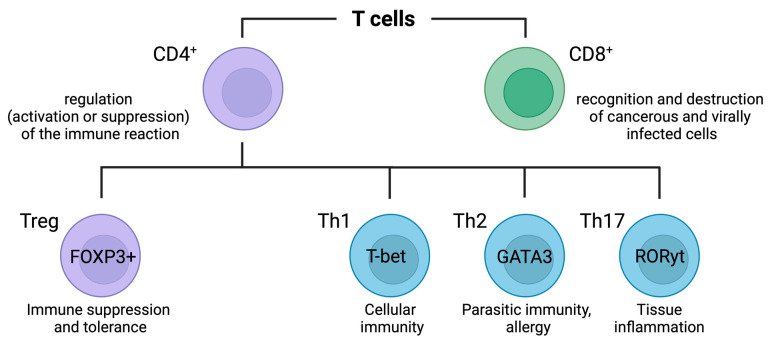
Overview of T cell subsets. CD8^+^ T cells, also known as cytotoxic T lymphocytes (CTLs), are a vital component of the adaptive immune system. Their primary function is to identify and destroy infected or malignant cells. CD4^+^ T cells, also known as helper T cells, are crucial for orchestrating the immune response. They assist other immune cells by releasing cytokines that regulate the activity, growth, and differentiation of various immune cells. CD4^+^ can further be divided into Th1 cells (expressing T-box expressed in T cells [T-bet]), Th2 cells (expressing the GATA binding protein 3 [GATA3]), Th17 cells (expressing the RAR-related orphan receptor gamma t [RORγt]), and T regulatory cells (Treg; expressing elevated levels of (Forkhead-Box-Protein P3 [FOXP3]). Th1 cells promote cell-mediated immunity by activating macrophages and cytotoxic T cells. They are crucial for defending against intracellular pathogens like viruses and certain bacteria. Th2 cells support humoral immunity by stimulating B cells to produce antibodies. They are important for combating extracellular parasites and allergens. Th17 cells are involved in defending against extracellular bacteria and fungi. They also play a role in autoimmune diseases by promoting inflammation. Tregs maintain immune tolerance by suppressing immune responses, preventing autoimmune diseases, and controlling inflammation. The figure was created with BioRender.com.

Upon activation of and exposure to lineage-specific cytokines, naive CD4^+^ T cells undergo differentiation into different lineages (Figure 4). This differentiation process is tightly linked with a metabolic shift towards aerobic glycolysis [72,74,75], with the regulation of glycolysis rates playing a pivotal role. An activation of CD4^+^ T cells promotes glucose uptake and aerobic glycolysis, influencing the differentiation of T cell subsets. Several glucose transporters (Glut), including Glut1, Glut3, Glut6, and Glut8, are upregulated and involved in this process [76]. The specific significance of inducing glucose import by increased Glut transporter expression in the differentiation of CD4^+^ T cells becomes particularly evident in cells with mutations or deletions in the genes for Glut1 and Glut3.

A deficiency in Slc2a1 (encoding the Glut1 protein) reduces thymocyte numbers, inhibits effector CD4^+^ T cell expansion, and diminishes cytokine production across various CD4^+^ T cell subsets. Notably, while Glut1 is dispensable for the lineage commitment of CD4^+^ Treg cells, the cell surface expression of Glut1 is high in Th1, Th2, and Th17 cells. Consistent with Glut1 expression patterns, Th1, Th2, and Th17 cells exhibit increased glycolysis rates, while Treg cells display increased rates of fatty acid oxidation [76,77].

Glut3 plays a crucial role in Th17 cell differentiation, as deficiency in Slc2a3 (encoding the Glut3 protein) inhibits this process [78]. Glut3-mediated glucose uptake is critical for mitochondrial glucose oxidation and subsequent acetyl-CoA production. In addition to its central function during energy production, acetyl-CoA plays a pivotal role in regulating the chromatin accessibility of loci associated with inflammatory genes, including IL17a [78].

Treg cells exhibit distinct glucose metabolic patterns compared to Th1, Th2, and Th17 cells, with significantly lower levels of Glut1 and reduced glycolytic rates [77].

In summary, it can be concluded that Th1, Th2, and Th17 cells exhibit elevated Glut levels and consequently an increased glycolytic flux. Conversely, Treg cells do not show an elevated glycolytic flux and tend to shift toward lipid metabolism.

#### 3.2.2. Increased Glucose Uptake in CD8^+^ T Cells

Similar to CD4^+^ T cells, the activation and differentiation of CD8^+^ T cells (Figure 4) are associated with an upregulation of Glut transporters [72]. However, there are characteristic differences. Unlike CD4^+^ T cells, CD8^+^ T cells do not rely on Glut1 for proliferation or granzyme B production. Instead, CD8^+^ T cells depend on Glut2 for optimal proliferation and effector cytokine production [72,76]. A deficiency in Slc2a2 (which encodes the Glut2 protein) affects CD8^+^ T cell activation [79]. Thus, Glut2 emerges as the primary factor involved in CD8^+^ T cell activation.

Increased glucose import via the induction of Glut transporters is a prerequisite for heightened glycolytic flux. Through enhanced glycolysis and the consequent increased activity of the TCA cycle, more reducing equivalents (NADH and FADH_2_) can be produced. By promoting glycolysis and channeling electrons through GPD2 to ubiquinone, RET is induced, enabling the generation of an oxidative signal from Complex I. The augmented electron influx into the ETC via the reducing equivalents NADH (Complex I) and FADH_2_ (Complex II) leads to the release of ROS at Complex III. Consequently, glucose metabolism is intricately associated with the generation of the oxidative signal.

### 3.3. Modulation of TCR Activation-Induced Signaling by Co-Stimulation

T cell activation is a complex process that requires multiple signals to ensure a robust and controlled immune response. TCR activation alone is not sufficient for complete T cell activation. Co-stimulatory signals are also required. Co-stimulatory receptors on T cells, such as CD28, the cytotoxic T lymphocyte-associated protein 4 (CTLA-4), and the programed cell death protein 1 (PD-1), interact with their respective ligands on APCs (e.g., CD80/CD86 for CD28 and CTLA-4, and PD-1L for PD-1). The balance between activating (e.g., CD28) and inhibitory (e.g., CTLA-4, PD-1) signals determines T cell activation, anergy, or exhaustion.

#### 3.3.1. CD28

CD28 is a critical co-stimulatory receptor on T cells that plays a vital role in their activation and survival. It works in tandem with the TCR to ensure a full and effective immune response. The interaction between CD28 on T cells and its ligands, CD80 and CD86, on antigen-presenting cells (APCs) is essential for the proper functioning of the immune system. Simultaneously to TCR stimulation, CD28 binds to its ligands CD80 or CD86 on the APC. This interaction provides a necessary secondary signal. The co-stimulatory signal from CD28 amplifies the TCR signal, leading to full T cell activation. Activation of CD28 initiates several intracellular signaling pathways, including the activation of PI3K/AKT, MAPK, and NF-κB pathways.

CD28 stimulation leads to the induction of ROS [8,80], which amplifies the oxidative signal induced by TCR activation. Unlike the mitochondrial origin of the TCR-derived oxidative signal, the CD28-depenent signal is generated by Arachidonate 5-Lipoxygenase (5-LO) [8,80,81].

#### 3.3.2. Cytotoxic T Lymphocyte-Associated Protein 4 (CTLA-4)

Cytotoxic T lymphocyte-associated protein 4 (CTLA-4) is an inhibitory receptor primarily expressed on activated T cells. Its main function is to downregulate immune responses, thereby maintaining immune homeostasis and preventing autoimmunity. CTLA-4 achieves this by binding to its ligands, CD80 and CD86, which are expressed on antigen-presenting cells. This binding competes with the costimulatory receptor CD28, which also binds to CD80 and CD86, but promotes T cell activation. By outcompeting CD28 for these ligands, CTLA-4 effectively inhibits T cell proliferation and cytokine production, leading to immune suppression [82].

The fact that CTLA-4 competes with CD80 and CD86 for binding explains its ability to block CD28-dependent ROS production via 5-LO. This was confirmed using a soluble CTLA-4-Ig protein, a chimeric protein consisting of the extracellular domain of human CTLA-4 and a fragment (hinge and constant region) of the Fc portion of human IgG1 [83]. CTLA-4-Ig successfully inhibited CD28-induced ROS production, thereby preventing T cell activation [84].

#### 3.3.3. Programmed Cell Death Protein 1 (PD-1)

Programmed cell death protein 1 (PD-1) is an inhibitory receptor expressed primarily on the surface of T cells, B cells, and myeloid cells. Its primary function is to regulate immune responses by maintaining self-tolerance and preventing autoimmunity. PD-1 achieves this by binding to its ligands, PD-L1 and PD-L2, which are often upregulated in various tissues, including tumor cells. This interaction inhibits T cell activation and proliferation, leading to reduced cytokine production and immune suppression. The PD-1/PD-L1 pathway is a critical mechanism for cancer cells to evade the immune system, making it a significant target for immunotherapy treatments.

Surprisingly, PD-1 stimulation or upregulation can generate ROS, probably through mitochondrial or mitochondria-related processes [85,86]. This ROS production is not associated with a proliferation or differentiation of T cells, but it is associated with the induction of apoptosis [85]. The CD95 death ligand (CD95L) is regulated by NFκB [56]. Naive or resting T cells are resistant to apoptosis, with CD95L acting as a weapon to induce apoptosis in neighboring (malignant or damaged) cells. Activated T cells become sensitive to apoptosis, where CD95L can induce fratricide in neighboring T cells or suicide within the same cell through being cleaved by the metalloprotease a disintegrin and metalloproteinase domain-containing protein 10 (ADAM10). This process, known as activation-induced cell death (AICD), is thought to eliminate autoreactive (persistently activated) T cells, thereby preventing autoimmunity [41,56,87,88,89].

## 4. Oxidative Signaling Modulates Signal Cascades and the Expression Profile of Activated T Cells

The oxidative signal can oxidize various proteins, including phosphatases, kinases, and transcription factors. This oxidation can either inhibit or induce protein activity and thereby modulate signal pathways.

### 4.1. Regulation of Phosphatases

The protein tyrosine phosphatases (PTPs) comprise a crucial group of redox-regulated proteins. Within their catalytic center, all PTPs have a redox-regulated cysteine. Oxidation of this thiol group by H_2_O_2_ leads to PTP inactivation. Given the reversibility of PTP oxidation, these proteins exist in two alternative states: an active state with a reduced cysteine or an inactive state with an oxidized cysteine [9,90]. ROS generated upon TCR stimulation rapidly convert to H_2_O_2_, which constitutes the primary oxidative signal. Thus, activation-induced oxidative signaling can inhibit PTPs, thereby regulating the signaling pathways they govern.

**Figure 5:**
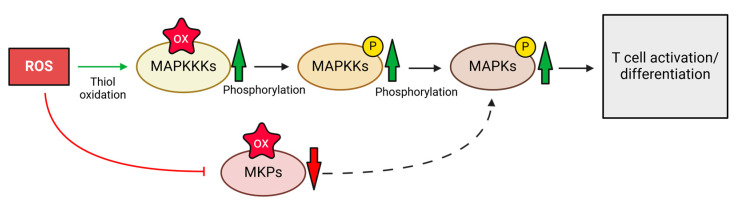
ROS-induced oxidation of free thiol groups activates MAP kinase kinase kinases (MAPKKKs), which phosphorylate MAPKK and, subsequently, MAPK. This signaling pathway is further amplified by the oxidation of MAPK phosphatases, which are inhibited by oxidation of a thiol residue in their active site, resulting in full activation of the MAPK cascade. OX = oxidation, P = phosphorylation. The figure was created with BioRender.com.

A particularly important signaling pathway in T cells is the mitogen-activated protein kinase (MAPK) pathway. These kinases are activated via phosphorylation and deactivated by dephosphorylation through MAPK phosphatases (MKPs). The activation-induced oxidative signal inactivates MKPs, thereby activating MAPKs (Figure 5) such as extracellular signal-regulated kinase (ERK), c-Jun N-terminal kinases (JNK), and p38, inducing cell proliferation and differentiation [90,91,92]. Another critical regulator of T cell activation and function is the Phosphatase and Tensin Homolog (PTEN). It acts as a tumor suppressor by dampening the Phosphoinositid-3-Kinase (PI3K)–Protein kinase B (PKB/AKT) signaling axis crucial for T cell activation, growth, and viability. Through its action of dephosphorylating phosphatidylinositol (3,4,5)-trisphosphate (PIP_3_), PTEN counteracts the PI3K pathway, thus restraining downstream AKT activation. This control aids in preserving T cell balance and preventing hyperactive immune reactions. ROS can oxidize PTEN at its active site cysteine residue, leading to its inhibition and therefore opening the AKT pathway, enabling T cell activation [93].

### 4.2. Regulation of Kinases

Kinases can be activated by oxidation. Again, the MAPK signaling pathway is the most prominent example in T cells. Activation of MAPKs typically involves a cascade of kinase reactions, progressing from MAP kinase kinase kinases (MAPKKKs) to MAP kinase kinases (MAPKKs), culminating in the final activation of MAPK [90,92,94]. ROS-induced oxidation of free thiol groups activates MAPKKKs, promoting kinase activity through conformational changes [95,96]. This, along with PTP inhibition, induces the MAPK signaling pathway, fostering T cell proliferation and differentiation (Figure 5).

### 4.3. Essential Transcription Factors for T Cell Activation

Oxidative signals not only activate or deactivate signaling pathways but also directly modulate the activity of transcription factors [97].

#### 4.3.1. Activator Protein-1 (AP-1)

The AP-1 transcription factors comprise homodimers and heterodimers formed by members of the Jun (c-Jun, JunB, JunD), Fos (c-Fos, FosB, Fra-1, Fra-2), Maf (c-Maf, MafA, MafB, MafG/F/K, Nrl), and ATF (ATF2, ATF3, B-ATF, JDP1, JDP2) protein subfamilies [90]. AP-1 dimers vary in composition, offering functional diversity. Different family members have unique affinities for DNA sequences and interaction partners crucial for the induction of processes like proliferation, differentiation, and apoptosis [98]. Numerous studies have demonstrated a highly conserved characteristic of AP-1 from yeast to mammals, namely its redox regulation and activation by oxidative signals [90]. For instance, exposure to ROS enhances both gene expression and protein levels of c-Fos and c-Jun, leading to increased DNA-binding activity in AP-1 [99,100]. In addition, ROS scavengers or antioxidants can effectively inhibit AP-1 activity [99,100,101,102]. The DNA-binding activity of c-Fos and c-Jun are determined by the redox state of several conserved free thiol groups. Many studies have shown that AP-1 is also indirectly regulated by ROS signals. The AP-1 family of transcription factors is a target for phosphorylation by the MAPK cascades, which, as described above, respond to ROS, leading to an enhanced transcriptional activation of AP-1 [29,90,91,98,101,103].

#### 4.3.2. Nuclear Factor Kappa B (NF-κB)

**Figure 6:**
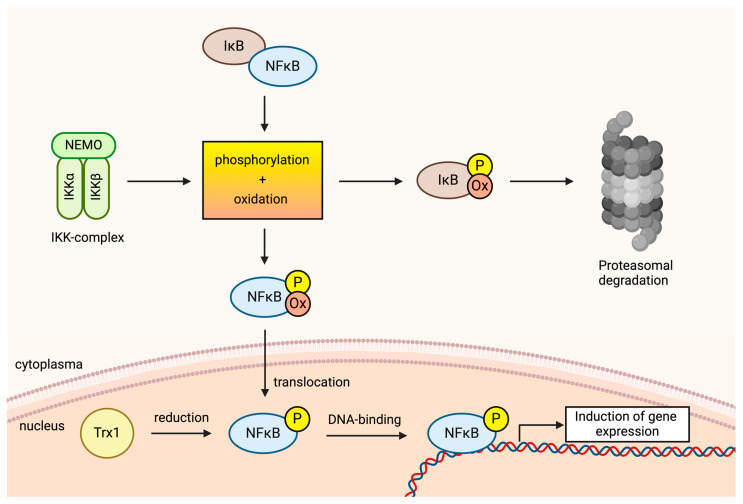
The role of oxidative signals in the activation of NF-κB. The inhibitor of NF-κB (IκB) is phosphorylated by the IκB kinase (IKK) complex. Additionally, a pro-oxidative environment in the cytosol causes IκB to become oxidized. Both phosphorylation and oxidation result in the accelerated degradation of IκB, so NF-κB is released. The free NF-κB can now translocate to the nucleus. Oxidation further accelerates this translocation. The oxidation of NF-κB is enabled by a shift in the cytosolic REDOX homeostasis towards a pro-oxidative state, induced by the oxidative signal. In the nucleus, NF-κB must be reduced again to enable optimal DNA binding. This is primarily achieved by Thioredoxin 1 (TRX1). The figure was created with BioRender.com 4.3.3. Nuclear Factor of Activated t cells (NF-AT).

NF-κB represents a collective term for dimeric transcription factors within the Rel family [90]. In the absence of activating signals, NF-κB is sequestered in the cytoplasm through binding to the inhibitor of NF-κB (IκB). Upon stimulation, the IκB kinase (IKK) complex is activated, phosphorylating IκB and marking it for ubiquitination and subsequent degradation (Figure 6). This degradation exposes the nuclear localization sequence of NF-κB, facilitating its translocation into the nucleus [104,105]. In the early 1990s, Schreck et al. demonstrated that treatment with H_2_O_2_ induces nuclear translocation [106]. Subsequent studies have revealed the inhibition of NF-κB activation by antioxidants [8]. Although H_2_O_2_ does not universally induce NF-κB activation across all cell types, it is generally recognized to be reliant on ROS in activated T cells [29,107]. Mechanisms of H_2_O_2_-induced NF-κB induction are diverse and dose-dependent. In the cytosol, a pro-oxidative environment prompts IκB oxidation and degradation, releasing NF-κB for nuclear translocation. Free thiol groups in NF-κB subunits are also oxidized in the cytosolic milieu, with thioredoxin (TRX)-1 primarily reversing this process in the nucleus (Figure 6). Notably, NF-κB must be in its reduced form in the nucleus to effectively bind to DNA binding sites [29,90,108,109].

NF-AT is primarily regulated by Ca^2+^**.** The increase in intracellular Ca^2+^ levels stimulated by TCR triggering activates the Ca^2+^-calmodulin-regulated phosphatase, calcineurin. Calcineurin, in turn, dephosphorylates the transcription factor NF-AT. Dephosphorylation exposes a nuclear localization signal, leading to the translocation of transcriptionally active NF-AT into the nucleus [56,110]. Additionally, it has been shown that ROS can also promote the nuclear translocation of NF-AT and enhance the transcriptional activity of NF-AT. However, the precise mechanisms involved in this process remain unclear [51].

#### 4.3.3. Nuclear Factor Erythroid 2-Related Factor 2 (NRF2)

The Keap1-Nrf2 signaling pathway is crucial for maintaining cellular redox equilibrium and protecting cells from oxidative stress. ROS plays a central role in this mechanism, dynamically balancing Nrf2 activation and inhibition by Kelch-like ECH-associated protein 1 (Keap1).

Under the conditions of a balanced redox equilibrium, Nrf2 is sequestered in the cytoplasm through its interaction with Keap1, which facilitates the ubiquitination and subsequent degradation of Nrf2. Upon the induction of oxidative stress, Keap1 releases Nrf2, allowing Nrf2 to stabilize and translocate into the nucleus. Once in the nucleus, Nrf2 initiates an antioxidative response, protecting cells from oxidative stress and the accumulation of cellular damage [90,111,112,113,114]. In particular, Nrf2 is involved in modulating the anti-inflammatory response by controlling redox balance and activating Antioxidant Response Enzyme (ARE)-mediated anti-inflammatory genes. This includes upregulating the expression of antioxidant genes like NQO1, HO-1, and PRX1, all of which possess anti-inflammatory properties [114].

On the one hand, a misregulated oxidative defense and the accumulation of ROS lead to extensive oxidative damage to macromolecules, including proteins, lipids, and DNA. Damage to proteins and lipids not only disrupts intracellular signaling but also leads to cell death. DNA oxidation results in the accumulation of mutations, which can cause the development of malignancies [111,115]. If regulation of the redox balance is disrupted due to alterations in the Nrf2/Keap1 signaling pathway, it can lead to increased oxidative damage and mutations, thereby raising the risk of tumor development [115,116,117,118]. On the other hand, Nrf2 protects cells by preventing the accumulation of ROS and thus oxidative stress. Tumors often exhibit elevated ROS levels due to their higher energy demands. In this context, the deregulation of Nrf2 and/or Keap1 is detrimental [118,119,120,121] as the induction of antioxidant genes by Nrf2 enables the survival of these malignant cells, which would otherwise die from oxidative damage. Additionally, resistance to chemotherapy increases because many chemotherapeutic agents work by shifting the redox balance and generating ROS [116,118,119]. Thus, Nrf2 is indeed a double-edged sword. It fine-tunes the redox balance, allowing oxidative signaling to occur. Additionally, it prevents the accumulation of mutations and the development of tumors by curbing excessive ROS production. In contrast, Nrf2 enables malignant cells to adapt to elevated ROS levels, thereby protecting them from cell death. Therefore, Nrf2 is a crucial factor in the regulation of the intracellular redox balance.

Taken together, ROS can oxidize essential signal proteins such as phosphatases, kinases, and transcription factors, altering their activity. Oxidation of specific functional amino acid residues within these proteins can either inhibit or enhance their function. Thus, oxidative signaling directly controls T cell proliferation and differentiation by impairing the activity of protein phosphatases, kinases, and transcription factors.

These ROS-dependent changes in signaling pathways and transcription factor activity significantly alter the expression profile of T cells. The minimal requirements for inducing IL2 expression, an essential factor in T cell activation, involve the activity of the transcription factors AP-1, NF-κB, and NF-AT. The MAP kinase cascade, primarily via ERK activation, significantly contributes to the induction of T cell proliferation and differentiation [29,35,122].

## 5. Conclusions

Redox signaling has evolved from being seen as a mere byproduct of enzymatic reactions to intentionally produced molecules crucial for cellular signaling. In T cells, ROS play a pivotal role in signaling pathways, with H_2_O_2_ acting as a vital second messenger. The precise localization of ROS generation and the balance between ROS and antioxidant defense mechanisms are critical for enabling oxidative signaling. Furthermore, cellular metabolic alterations are essential for generating oxidative signals, emphasizing the intricate interplay between metabolism and signaling in T cell activation, proliferation, and differentiation. Overall, redox signaling is indispensable for fine-tuning cellular responses, particularly in the context of T cell function and immune responses [29,35].

**Figure 7:**
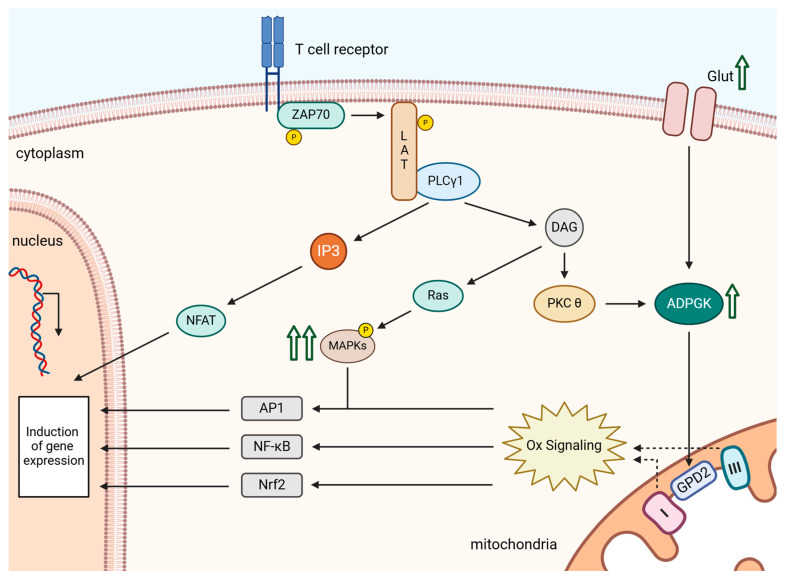
After TCR stimulation, two signaling pathways are induced via 3,4,5-triphosphate (IP_3_) and diacylglycerol (DAG). The IP_3_-dependent pathway leads to Ca^2+^ release into the cytosol, activating NF-AT. The DAG-dependent pathway activates PKCθ and induces an oxidative signal via Complex I and III of the mitochondrial electron transport chain (ETC). Additionally, the MAPK signaling pathway is induced via PKCθ. The oxidative signal originating from mitochondrial ETC amplifies the MAPK signaling pathway and activates the redox-sensitive transcription factors AP1, NF-κB, and Nrf2. AP1 and NF-κB, together with NF-AT, create the minimal requirement for T cell activation. Conversely, Nrf2, by inducing antioxidant proteins, contributes to the control and potential termination of the oxidative signal. The figure was created with BioRender.com Oxidative signals in T cells are essential for the initiation of a T cell immune response. Several publications and data have addressed the induction of oxidative signals by TCR stimulation and the mechanisms by which these signals are generated. However, the role of co-stimulation in oxidative signaling is not well understood. This represents a knowledge gap that urgently needs to be investigated in detail.

Stimulation of the TCR by an antigen triggers activation of the proximal T cell machinery, leading to activation of the tyrosine kinase ZAP70. ZAP70 phosphorylates the scaffold protein LAT, which in turn recruits proteins like PLCγ1. Recruitment to LAT activates PLCγ1, leading to the production of IP_3_ and DAG. IP_3_ then induces the opening of calcium channels in the endoplasmic reticulum (ER). This initial influx of Ca^2+^ is followed by a stronger influx through calcium channels in the plasma membrane. This increase in cytosolic Ca^2+^ levels represents one arm of the TCR signaling pathway. The other arm of TCR signaling involves DAG. DAG activates PKC, with a subpopulation of PKCθ translocating to the outer mitochondrial membrane. Additionally, PKCθ likely phosphorylates ADPGK, inducing increased glucose influx and a metabolic switch. This results in an electron transfer to the CytQ pool, directly mediated by GPD2. At this point, hyper-reduced ubiquinol likely leads to electron flow in the ETC retrograde to Complex I. During this process, ROS are released into the mitochondrial matrix. Simultaneously, electrons may also flow forward from ubiquinol to Complex III, which then releases ROS into the intermembrane space. The release of ROS via Complexes I and III of the ETC constitutes the oxidative signal [36,51]. The Ca^2+^ signal activates the transcription factor NF-AT via calcineurin, an integral component of T cell activation. The oxidative signal enhances/enables the activation of kinase signaling cascades through the modification/oxidation of kinases and regulatory phosphatases (e.g., MAPK signaling cascade). Additionally, REDOX-sensitive transcription factors such as AP-1 and NF-κB are also modified/oxidized, thereby increasing their translocation into the nucleus. The oxidative signal also induces the transcription factor Nrf2. Nrf2 regulates a variety of antioxidant proteins, which then prevent an excess of ROS production, thus regulating/terminating the oxidative signal. The transcription factors NF-AT, AP-1, and NF-κB form the minimal requirement for T cell activation. Thus, the Ca^2+^ signal in combination with the oxidative signal is indispensable for the activation of T lymphocytes [8,29,35,36,51,90] (Figure 7).

Changes in the redox balance and oxidative signals are ubiquitous and play a fundamental role in both inter- and intracellular communication. In the case of T cell activation, these signals are indispensable. A more in-depth exploration of these signals, their formation, and the impact of metabolic changes on these signals or, conversely, how changes in the redox equilibrium affect metabolism, is imperative and requires further investigation.

The role of the activating co-stimulatory surface molecule CD28 was last studied in detail in the late 1990s [8]. New investigative methods could provide more precise insights into the production of oxidative signals through CD28 stimulation and elucidate how these signals interact.

CTLA-4, which competes with CD28 for its ligands, attenuates T cell activation. This competition naturally reduces the oxidative signal that can be induced by CD28. This has potential clinical applications; soluble CTLA-4 can be used to dampen T cell immune responses, thereby preventing excessive immune responses and autoimmunity [83,84]. However, further research is needed to investigate in detail how CTLA-4 stimulation affects TCR-induced oxidative signaling.

For PD-1, the situation is more complex. There are limited data on the generation of oxidative signals or changes in redox balance. The available data suggest that PD-1 stimulation initiates an oxidative signal. This is puzzling because oxidative signals contribute to T cell activation, whereas PD-1 limits or inhibits T cell activation [85,86]. However, oxidative signals are also crucial for terminating an immune response by inducing CD95L, which can trigger apoptosis (AICD). Naive and resting T cells are resistant to apoptosis. If CD95L is induced, these cells survive. In contrast, activated T cells are sensitive to apoptosis, and CD95L induction leads to apoptosis (AICD) in these cells [56,88]. The ability of oxidative signals in T cells to terminate an immune response is crucial for preventing autoimmune reactions. 

However, induction of AICD can be dysregulated in certain diseases. In Human Immunodeficiency Virus (HIV) infection, the trans-activating regulatory protein (Tat) induces an oxidative signal, sensitizing T cells to AICD [41,123,124]. This may be a mechanism that leads to T cell depletion following HIV infection and the development of the acquired immune deficiency syndrome (AIDS). This demonstrates how little we know about oxidative signals and highlights the importance of pursuing further research in this area.

However, information on how oxidative signals affect transcription factors in T cells has already been exploited clinically. In cutaneous T cell lymphoma (CTCL), especially in its incurable and aggressive leukemic variant, the Sézary syndrome, there is constitutive NF-κB activation. This leads to increased proliferation and apoptosis resistance in malignant T lymphocytes. In the cytosol, a pro-oxidative environment promotes NF-κB translocation to the nucleus. Within the nucleus, however, an anti-oxidative environment is required for NF-κB to be reduced and able to bind to DNA. The reduction in NF-κB is primarily mediated by Trx-1. When Trx-1 is inhibited in the nucleus, NF-κB can no longer be reduced, leading to the downregulation of important protective NF-κB target genes, such as inhibitors of apoptosis (IAPs). As a result, a cytosolic death platform known as the ripoptosome can form, inducing apoptosis and necroptosis in malignant cells [108]. Trx-1 can be specifically inhibited by the small molecule dimethyl fumarate (DMF). Treatment with DMF has been shown to significantly induce cell death in CTCL cells, both in vitro and in vivo. Healthy T cells were spared by DMF as they do not require constitutive NF-κB activation for survival [108,125,126]. These findings were also corroborated in a phase II clinical study [127].

These studies underscore the importance of understanding oxidative signals—their origins, mechanisms of action, and how they can be modulated. A precise understanding of the redox balance will allow us to modulate immune responses, understand disease, and develop new treatments.
